# Clinicopathologic study of intestinal spirochetosis in Japan with special reference to human immunodeficiency virus infection status and species types: analysis of 5265 consecutive colorectal biopsies

**DOI:** 10.1186/s12879-014-0736-4

**Published:** 2015-01-13

**Authors:** Yoko Tateishi, Masae Takahashi, Shin-ichiro Horiguchi, Nobuaki Funata, Koichi Koizumi, Koji Okudela, Tsunekazu Hishima, Kenichi Ohashi

**Affiliations:** Department of Pathology, Yokohama City University Graduate School of Medicine, Yokohama, Japan; Department of Pathology, Tokyo Metropolitan Cancer and Infectious Disease Center Komagome Hospital, Tokyo, Japan; Department of Internal Medicine, Tokyo Metropolitan Cancer and Infectious Disease Center Komagome Hospital, Tokyo, Japan

**Keywords:** Intestinal spirochetosis, Human Immunodeficiency Virus (HIV) infection, Colorectal biopsies, *Brachispira pilosicoli*, *Brachyspira aalborgi*

## Abstract

**Background:**

Previous studies reported that the incidence of intestinal spirochetosis was high in homosexual men, especially those with Human Immunodeficiency Virus infection. The aim of the present study was to clarify the clinicopathological features of intestinal spirochetosis in Japan with special reference to Human Immunodeficiency Virus infection status and species types.

**Methods:**

A pathology database search for intestinal spirochetosis was performed at Tokyo Metropolitan Cancer and Infectious Disease Center Komagome Hospital between January 2008 and October 2011, and included 5265 consecutive colorectal biopsies from 4254 patients. After patient identification, a retrospective review of endoscopic records and clinical information was performed. All pathology slides were reviewed by two pathologists. The length of the spirochetes was measured using a digital microscope. Causative species were identified by polymerase chain reaction.

**Results:**

Intestinal spirochetosis was diagnosed in 3 out of 55 Human Immunodeficiency Virus-positive patients (5.5%). The mean length of intestinal spirochetes was 8.5 μm (range 7–11). *Brachyspira pilosicoli* was detected by polymerase chain reaction in all 3 patients. Intestinal spirochetosis was also diagnosed in 73 out of 4199 Human Immunodeficiency Virus-negative patients (1.7%). The mean length of intestinal spirochetes was 3.5 μm (range 2–8). The species of intestinal spirochetosis was identified by polymerase chain reaction in 31 Human Immunodeficiency Virus-negative patients. *Brachyspira aalborgi* was detected in 24 cases (78%) and *Brachyspira pilosicoli* in 6 cases (19%). Both *Brachyspira aalborgi* and *Brachyspira pilosicoli* were detected in only one Human Immunodeficiency Virus-negative patient (3%). The mean length of *Brachyspira aalborgi* was 3.8 μm, while that of *Brachyspira pilosicoli* was 5.5 μm. The length of *Brachyspira pilosicoli* was significantly longer than that of *Brachyspira aalborgi* (*p* < 0.01). The lengths of intestinal spirochetes were significantly longer in Human Immunodeficiency Virus-positive patients than in Human Immunodeficiency Virus-negative patients (*p* < 0.05).

**Conclusions:**

The incidence of intestinal spirochetosis was slightly higher in Human Immunodeficiency Virus-positive patients than in Human Immunodeficiency Virus-negative patients. However, no relationship was found between the Human Immunodeficiency Virus status and intestinal spirochetosis in Japan. *Brachyspira pilosicoli* infection may be more common in Human Immunodeficiency Virus-positive patients with intestinal spirochetosis than in Human Immunodeficiency Virus-negative patients with intestinal spirochetosis.

## Background

**Figure 1:**
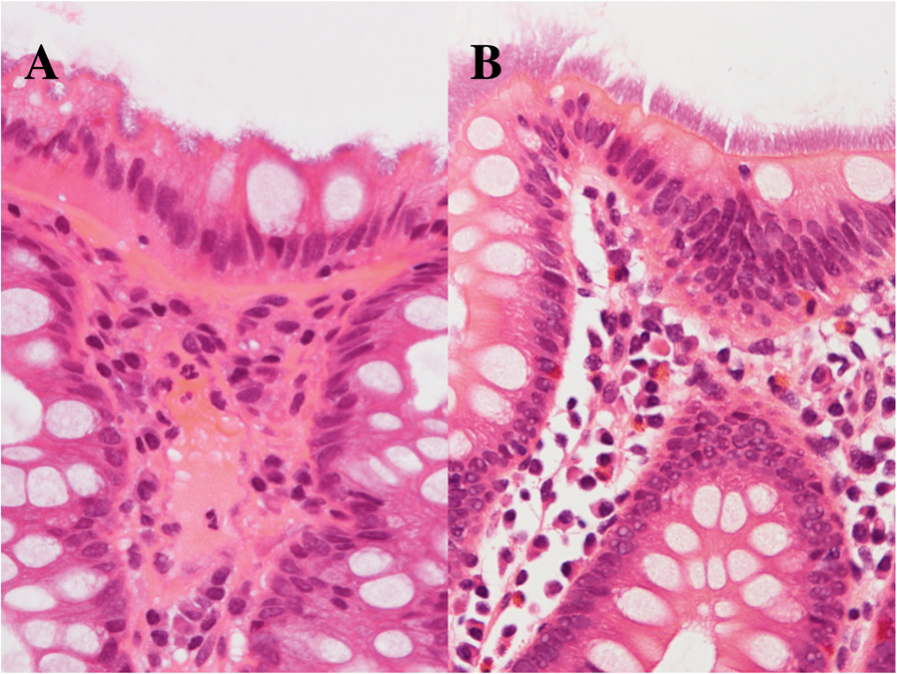
**Microscopic appearance of intestinal spirochetosis.** (H&E staining) **A** *Brachyspira aalborgi*. Basophilic fringes measuring 2 to 6 micrometers in length were observed on the surface epithelium. **B** *Brachyspira pilosicoli.* Basophilic fringes measuring 4 to 20 micrometers in length were observed on the surface epithelium.

Intestinal spirochetosis (IS) is a condition in which colonic and appendiceal epithelial cells are colonized by one of two anaerobic spirochetes, either *Brachyspira aalborgi,* measuring 2 to 6 μm in length, or *Brachyspira pilosicoli*, measuring 4 to 20 μm in length (Figure 1) [1,2]. These two species may be zoonotic because they have previously been isolated from the feces of non-human primates and other animals [3]. However, it has yet to be determined whether IS is a pathogen or commensal inhabitant [4-6].

Previous studies reported that the incidence of IS was high in homosexual men, especially those with Human Immunodeficiency Virus (HIV) [7-10]. Although IS is generally considered to be associated with immunocompromised states such as HIV infection, it has also been detected in patients with a normal immune status [5,11-13]. The incidence of IS in HIV-positive patients has not yet been reported in Japan. Furthermore, the prevalence of these species in HIV-positive patients remains unclear [11,13-15]. Therefore, the aim of the present study was to clarify the clinicopathological features of IS in Japan with special reference to HIV infection status and species types.

## Methods

This study was approved by the Research Ethics Committees at the Tokyo Metropolitan Cancer and Infectious Disease Center Komagome Hospital, and performed according to its guidance. Informed consent was obtained from all participants. A pathology database search for IS was performed at the Tokyo Metropolitan Cancer and Infectious Disease Center Komagome Hospital between January 2008 and October 2011, and included 5265 consecutive colorectal biopsies from 4254 patients. Patients consisted of 2558 men and 1696 women, whose age ranged between 9 and 93 years with a mean age of 64 years. Fifty-five of these patients were HIV-positive (1.3%) and 4199 were HIV-negative (98.7%). After patient identification, a retrospective review of endoscopic records and clinical information was performed.

The database search identified 76 patients with IS. All pathology slides were collected and reviewed by two pathologists (YT and NF). All specimens were stained with hematoxylin and eosin and examined under X 400 magnification. IS was identified by characteristic basophilic fringes on the surface epithelium [13]. Immunostaining for spirochetosis was performed an antibody against *Treponema pallidum* (polyclonal, dilution 1:300; Concord CA, Biocare Medical) where necessary to confirm or refute the diagnosis. The lengths of spirochetes were measured using a digital microscope (Leica DMD 108).

Causative species were identified by PCR, which amplified the species-specific portion of the 16S ribosomal RNA gene. DNA from paraffin-embedded samples was extracted using NucleoSpin Tissue (MACHEREY-NAGEL, Duren, Germany). Pairs of primers were designed to detect the genes for 16S ribosome RNA (16S rRNA) and NADH oxidase (nox) in *B. aalborgi* and *B. pilosicoli* according to the method described by Mikosza *et al*. [16]. The integrity of paraffin-derived DNA was evaluated by PCR for D8S348. The sequence of primers and size of products are listed in Table 1.

**Table 1:** **Sequences of primers to detect the gene for 16S rRNA, NADH oxydase (nox) of** ***Brachyspira aalborgi and Brachyspira pilosicoli***

**Primer**	**Sequence**	**Size of products (bp)**
*B. aalborgi* 16S rRNA	F: TACCGCATATACTCTTGAC	472
	R: CCTACAATATCCAAGAACC	
*B. aalborgi* nox	F: GGTTGACTCAAGCACTAC	334
	R: AAACCGTATTTTGTTCCAGG	
*B. pilosicoli* 16S rRNA	F: AGAGGAAAGTTTTTTCGCTTC	196
	R: GTCGCTCCATCAGACTTT	
*B. pilosicoli* nox	F: GTAACTCCTCCTATTGAG	465
	R: GCACCATTAGGTAAAGTC	
D8S348	F: ACCGACAGACTCTTGCCTCCAAA	408
	R: TCACTCAGCTCCCATAACTTGGCAT	

F, forward; R, reverse.

All statistical analyses were performed using the JMP statistics software package (SAS; version 9.0.2). The relationship between IS and clinicopathological characteristics was assessed. Comparisons between two groups were analyzed with the Chi-square test. Differences were considered significant when the probability value was < 0.05.

## Results

IS was detected in 76 out of 4254 patients (1.7%) by hematoxylin and eosin staining. Immunostaining for spirochetosis was performed to confirm this diagnosis in 8 cases. No cases were excluded by spirochetosis immunostaining. Patients with IS consisted of 58 men and 18 women, whose age ranged from 30 to 83 years with a mean age of 59.3 years. IS was diagnosed in 3 out of 55 HIV-positive patients (5.5%), and 73 out of 4199 HIV-negative patients (1.7%). A correlation was found between IS and sex (*p* < 0.01) and age (*p* < 0.01) (Table 2). No significant difference was found between the HIV status and IS (*p* = 0.09).

**Table 2:** **Relationship between Clinicopathological Findings and IS**

	**IS positive**	**IS negative**	
	**n = 76**	**n = 4178**	
Mean age, y (range)	59.3 (30–83)	64.1 (9–93)	*p* < 0.01
Sex			
Men (n = 2558)	58 (2.3%)	2500 (97.7%)	*p* < 0.01
Women (n = 1696)	18 (1.1%)	1678 (98.9%)	
HIV			
Positive (n = 55)	3 (5.5%)	52 (94.5%)	*p* = 0.09
Negative (n = 4199)	73 (1.7%)	4126 (98.3%)	

IS was identified by characteristic 2–11 -μm-thick basophilic fringes on the luminal surface of the epithelium. The mean lengths of IS were 8.5 μm in HIV-positive patients and 3.5 μm in HIV-negative patients. The length of IS was significantly longer in HIV-positive patients than in HIV-negative patients (*p* < 0.05).

The identification of species by PCR amplification was assessed in 76 cases. Products of the gene for 16S rRNA in *B. aalborgi* were detected in 24 cases. Products of the gene for 16S rRNA in *B. pilosicoli* were detected in 9 cases. Both products of the genes for *B. aalborgi* and *B. pilosicoli* were detected in 1 case. The integrity of paraffin-derived DNA was evaluated by PCR for D8S348 in 42 cases which were negative for *B. aalborgi* and *B. pilosicoli*. In 31 out of 42 cases, D8S348 was not amplified suggesting the quality of DNA was insufficient. In remaining 11 cases, DNA extracted from IS in infected human tissue samples may be too small in relative amount or too poor in quality. Another possibility is these cases may have contained other species of spirochetes that have yet to be characterized [17]. The length of IS was shown in Table 3. The mean length of *B. aalborgi* was 3.8 μm (range 2–4.5) and the mean length of *B. pilosicoli* was 5.5 μm (range 3.2-11). The length of IS was 2.5 μm in patients with *B. aalborgi* and *B. pilosicoli*. The length of *B. pilosicoli* was significantly longer than that of *B. aalborgi* (*p* < 0.01).

**Table 3:** **Relationship between size of IS and species identified by PCR analysis**

**Size of IS**	**Case of** ***B. aalborgi***	**Case of** ***B. pilosicoli***	**Case of both** ***B. aalborgi*** **and** ***B. pilosicoli***
**(μm)**	**n = 25**	**n = 9**	**n = 1**
0<	1	0	0
2<	11	0	1
3<	11	4	0
4<	2	2	0
6<	0	1	0
8<	0	2	0

*p* < 0.01.

*B. pilosicoli* was detected in all 3 IS cases with HIV (100%). In HIV-negative patients, *B. aalborgi* was detected in 24 cases (78%) and *B. pilosicoli* was detected in 6 cases (19%). Both *B. aalborgi* and *B. pilosicoli* were detected in only one HIV-negative patient (3%). All 3 HIV-positive IS patients were male, not severely immunosuppressed (CD4 lymphocyte cells; 668/μL, 798/μL and 317/μL), had no other HIV-associated complications, showed no histological abnormalities other than IS, and had watery diarrhea**.** Most HIV-negative IS patients had no clinical symptoms. Forty-seven patients underwent a screening exam. Twelve patients underwent colonoscopy as a hemoccult-positive rectal examination. Two patients had hemorrhoids and one had ulcerative colitis. Eleven HIV-negative IS patients had clinical symptoms (15%); 6 had abdominal pain, 4 had diarrhea or loose stools, and 1 had abdominal pain and diarrhea. A correlation was found between the clinical symptoms and HIV status of IS patients (*p* < 0.01). The characteristics of IS patients with clinical symptoms were shown in Table 4. IS patients with clinical symptoms consisted of 10 men and 4 women, whose age ranged from 43 to 73 years with a mean age of 57.1 years. Endoscopically, tubular adenoma, or sessile serrated adenoma or adenocarcinoma was detected in 10 out of 14 cases, whereas no specific endoscopic findings were found in 4 out of 14 cases. The mean length of IS was 4.5 μm (range 2.5-11). *B. pilosicoli* was detected in all 3 HIV-positive patients. Each species was successfully identified in 6 out of 11 HIV-negative IS cases with clinical symptoms. *B. aalborgi* was detected in 3 cases and *B. pilosicoli* was detected in 2 cases. Both *B. aalborgi* and *B. pilosicoli* were detected in 1 case.

**Table 4:** **Characteristics of IS patients with clinical symptoms**

**Case**	**Ages as ranges**	**Clinical Symptoms**	**Endoscopic findings**	**Length of IS (μm)**	**Results of PCR**
1	55-59	Abdominal pain, diarrhea	TA	3	A
2	60-64	Abdominal pain	TA	4.2	P
3	70-74	Abdominal pain	TA, HP	4	ND
4	50-54	Abdominal pain	TA	3	A
5	60-64	Abdominal pain	ADC	2.5	ND
6	45-49	Abdominal pain	TA	3.5	ND
7	55-59	Abdominal pain	Normal	4	P
8	50-54	Diarrhea	TA	3	A
9	60-64	Diarrhea	TA	4	ND
10	70-74	Loose stool	SSAP	3	A
11	60-64	Loose stool	HP, TA	2.5	A,P
12	45-49	HIV+, diarrhea	Normal	8	P
13	40-44	HIV+, diarrhea	Normal	11	P
14	45-49	HIV+, diarrhea	Normal	7	P

F, female; M, male; TA, tubular adenoma; HP, hyperplastic polyp; SSAP, sessile serrated adenoma/polyp; ADC, adenocarcinoma; A, *B. aalborgi*; P, *B. pilosicoli;* ND, not detected.

## Discussion

The prevalence of IS was previously reported to be between 2 and 7% in Western countries and 11-34% in less developed countries [18,19]. Our study revealed that the incidence of IS in Japan was 1.7%, which was similar to that in Western countries. The prevalence of IS was previously reported to be approximately 54% in men who have sex with men (MSM) and HIV-positive patients [9,18,20]. On the other hand, Orenstein *et al.* identified four patients with IS among 82 HIV-positive patients [21]. Our study revealed that the incidence of IS was slightly higher in HIV-positive patients than in HIV-negative patients; however, no correlation was found between the HIV status and IS in Japan. Fifty-five HIV-positive and 4199 HIV-negative patients were evaluated in our study, which was one of the largest reported series of IS in HIV-positive and HIV-negative patients at a single institution.

Previous studies showed that the length of *B. aalborgi* was 2 to 6 μm and *B. pilosicoli* was 4 to 20 μm [1,2]. In patients in which the species was identified by PCR amplification, the length of *B. aalborgi* was 2 to 4.5 μm and *B. pilosicoli* was 2.5 to 11 μm. The length of *B. pilosicoli* was significantly longer than that of *B. aalborgi* (*p* < 0.01), which was consistent with the findings of previous studies. Furthermore, the length of IS in HIV-positive patients was significantly longer than that of IS in HIV-negative patients (*p* < 0.05).

*B. aalborgi* has been confirmed as the most prevalent species in Western countries and Japan, while *B. pilosicoli* is rare [6,13]. In the present study, *B. pilosicoli* was detected in all 3 HIV-positive patients. The results of the present study suggest that *B. pilosicoli* infections were more common in HIV-positive patients with IS while *B. aalborgii* infections may be more common in HIV-negative patients with IS in Japan. The prevalence of these species in HIV-positive patients has not yet been examined in detail [11,13-15]. To the best of our knowledge, this is the first study to show that *B. pilosicoli* infections may be more common in HIV-positive patients with IS based on one of the largest series composed of HIV-positive and HIV-negative patients examined at a single institution.

All 3 HIV-positive patients with IS in this study were male, less severely immunosuppressed, and had watery diarrhea. Anthony et al. reported that three HIV-positive IS patients were symptomatic with diarrhea [22]. A previous study demonstrated that IS caused chronic diarrhea in HIV-positive men who have sex with men, but were not severely immunosuppressed (CD4 lymphocyte cells >200/ μL) [20]. The clinicopathological findings of IS cases in our study were similar to these findings.

*B. aalborgi* is generally considered to be a non-pathogenic commensal, while *B. pilosicoli* is an opportunistic pathogen [23]. All three HIV-positive patients with *B. pilosicoli* in our study had watery diarrhea. In HIV-negative patients, *B. pilosicoli* was more commonly detected in patients with clinical symptoms (50%) than in patients without clinical symptoms (16%). Furthermore, the prevalence of *B. pilosicoli* was markedly higher in patients with clinical symptoms than in patients without clinical symptoms (*p* = 0.02). This result supports the pathogenic potential of *B. pilosicoli*. Since the number of patients was too small to clarify the clinical significance of *B. pilosicoli* infection, further investigations are required.

## Conclusion

In this study, we revealed the clinicopathological features of IS in Japan. The incidence of IS was slightly higher in HIV-positive patients than in HIV-negative patients. However, no relationship was found between the HIV status and IS. *Brachyspira pilosicoli* infection may be more common in HIV-positive patients with IS than in HIV-negative patients with IS.
